# On the Nature of Tubercles—Phthisis Pulmonalis

**Published:** 1829-04-01

**Authors:** 


### NATURE OF TUBERCLES—PHTHISIS PULMONALIS.

The following letter, signed "MEDICUS," and dated from Exeter, is inserted, because it relates immediately to an important practical subject, involving the health and lives of a great proportion of the population of this country. We may be allowed, once more, to protest against the habit of identifying the Editor of this Journal with every opinion that may appear in the various reviews of books. The review of Dr. Alison's work was written by a talented physician, but not by the Editor. This, however, is of little consequence.

SIR,—Having perused Dr. Alison's observations on that doubtful question, *the origin of tubercles*, and your remarks upon them, is my apology for entering upon a subject so involved in mystery ; lest the adoption of his opinions, sanctioned by you, give currency to the practice of indiscriminate depletion and starvation, which, in a majority of cases, will shorten the duration of human life. I do not mean to deny that cases may, and do occur, which may render early temperate depletion necessary, to control *active* inflammatory action ; but, from my own experience, I am led to believe that the greater number of cases are to be met in habits of lax fibre, in which the digestive organs were *primarily* invaded, be the cause what it may. That a foreign body ("quicksilver") should excite active inflammatory action in the pulmonary organs is a natural consequence ; the same effect is produced in all organized tissues, until the extraneous substance be removed by the vis medicatrix, or the interference of art ; but to say that tubercles, thus generated, are vested with the distinctive attributes of tubercles found in the scrofulous subject, is a doctrine I can by no means subscribe to, unless Dr. A. is prepared to prove an *identity* in their constituent principles ; in the latter, chemical analysis has detected phosphate and carbonate of lime, but, I apprehend, such result could not be discovered in tubercles created by *artificial* means. In the one, by removing the *cause* of inflammatory action, the *effect* will soon cease—not so in the other ; for, after inflammatory action has been subdued by depletion and antiphlogistic treatment, the disease will progress to the softening stage, under the *first* exciting cause. Again; in the one case, the inflammation will be *active*, and disorganization will be early induced; while, in the other the inflammation is *passive*, and tardy in its advance, such as we would expect to meet with in those habits in which they are found. Dr. A. seems, also, to have overlooked the fact, that tubercles in the lungs differ from tubercles in the other viscera, being, in their incipient stage, more vesicular, and generally met with at the *extremities* of the bronchial tubes. In this hasty sketch, which I will hereafter pursue at greater length, I am induced to offer my views on the subject, with the two-fold object of guarding against the continuance of a treatment which I have found highly injurious, (repeated depletion) and with the hope to reconcile, in some degree, the existing discrepancy of opinion upon a question which involves human life. The matter of tubercle I consider to be a peculiar morbid secretion, preceded by active congestion in the invaded organs, and that congestion may, in one case, be the effect of languid circulation in the exhalent arteries, as a consequence of debility; while, in another case, it may be the result of *passive* inflammatory action, dependent upon idiosyncrasy.

Placed, as I am, in the centre of our pulmonary depôts, I have abundant opportunities for observation, and a variety of interesting cases will form the subject of future communications.

Medicus.

---

